# Novel and potential future therapeutic options in Sjögren's syndrome

**DOI:** 10.1016/j.heliyon.2024.e38803

**Published:** 2024-10-01

**Authors:** Ting Zhao, Runrun Zhang, Zhaofu Li, Dongdong Qin, Xinchang Wang

**Affiliations:** a Key Laboratory of Traditional Chinese Medicine for Prevention and Treatment of Neuropsychiatric Diseases, Yunnan University of Chinese Medicine, Kunming, 650500, China; b The Second Clinical Medical College, Zhejiang Chinese Medical University, Hangzhou, 310000, China

**Keywords:** Sjögren's syndrome, Targeted therapies, Drugs, Immune, Cutting-edge treatment technologies

## Abstract

Sjögren's syndrome (SS) is a chronic autoimmune disease affecting the exocrine glands and can lead to various systemic symptoms impacting multiple organs. Despite its common occurrence, treatment options for SS have been largely limited, primarily focusing on alleviating symptoms rather than addressing the underlying autoimmune causes. A shift towards personalized medicine leads to the development of new therapeutic strategies aimed at targeting specific molecular pathways implicated in SS. Innovations in biologics are paving the way for inhibiting particular cytokines or cell surface molecules directly involved in the autoimmune mechanism. Furthermore, advancements in regenerative medicine, including the promising field of stem cell therapy, offer the potential for restoring or replacing the impaired salivary and lacrimal glands, providing hope for a more permanent resolution to this condition. This review encompasses cutting-edge treatment strategies for SS, spanning clinical and preclinical drugs to the latest treatment technology. Such advancements promise to drive targeted therapy development and inspire innovative ideas for treatment paradigms in SS.

## 1 Introduction

Sjögren's syndrome (SS) is a complex autoimmune disorder that primarily affects the salivary and lachrymal glands, generally causing a typical dryness of the eyes and the mouth. The disease is characterized by B-cell polyclonal activation and the production of autoantibodies, and it presents with various clinical symptoms. Non-Hodgkin lymphoma is a severe complication of SS. It occurs in 5–10 % of patients [1,2]. In severe cases, the extra-glandular manifestations can be life-threatening [3,4]. Therefore, SS is a major focus area in research on rheumatic and autoimmune diseases.

The pathogenesis of SS involves a complex interaction of environmental and endogenous factors. Autoantigens are recognized by antigen-presenting dendritic cells, leading to T and B cell activation, cytokine release, autoantibody generation by plasma cells, formation of ectopic germinal centers, and damage to the salivary gland epithelial cells [5]. The development of SS also involves adaptive immune responses, including the activation of T and B cells. This activation is tightly regulated by co-stimulatory pathways such as CD80/CD86: CD28, CD40: CD40L, and ICOS: ICOS-L, as well as co-inhibitory signals like CTLA-4. Additionally, the innate immune responses are associated with SS pathogenesis. Toll-like receptors (TLRs) are essential in mediating inflammatory pathways mainly involved in innate immunity. The nuclear transcription factor kappa B (NF-κB) is a critical player in the downstream signaling pathway of TLRs. Once activated, NF-κB can enter the nucleus to regulate the expression of inflammatory cytokines [6]. Moreover, TLRs are the primary pattern recognition receptors in the pathway of type I interferons (IFN-Is). IFN-Is may activate the Janus kinase/signal transducer and activator of the transcription (JAK-STAT) pathway and induce the expression of inflammatory genes [7]. Inhibition of the JAK-STAT pathway may suppress the expression of the B-cell activating factor belonging to the TNF family (BAFF, also termed BLyS) in SS [8]. Activation of pattern recognition receptors through the downstream pathway can directly lead to organ inflammation and is associated with activating adaptive immune responses [9]. During immune cell activation, pro-inflammatory factors are released, leading to sustained and persistent inflammatory responses, amplifying tissue damage, and causing progressive functional damage to affected organs.

Current SS treatment recommendations emphasize managing sicca symptoms and stimulating residual glandular function using muscarinic agonists (pilocarpine, cevimeline) as well as using conventional synthetic disease-modifying antirheumatic drugs (hydroxychloroquine, methotrexate, sulfasalazine, and leflunomide) and biological therapies (RTX, abatacept, and belimumab) for treating specific organ complications [10,11]. Saliva substitutes and artificial tears are topical medications commonly used to alleviate dryness symptoms associated with SS, but medications cannot inhibit disease progression [12,13]. Systemic medications such as glucocorticoids and immunosuppressive drugs are recommended for SS patients with systemic involvement, according to the EULAR recommendations. For SS patients without systemic involvement, it is generally recommended to use only topical medications to alleviate dryness symptoms. While some medications have shown mild benefits, traditional systemic immunosuppressive therapy has not been proven to effectively control the symptoms of syndrome sicca [14]. Similarly, biological therapies have not shown significant clinical effects in SS [5,15]. Emerging therapeutic strategies are exploring a more personalized medicine approach that targets specific molecular pathways involved in the SS. These strategies include novel drugs directly inhibiting specific cytokines or cell surface molecules involved in the autoimmune process. Strategies for treatment focus on various components such as B cells, inflammatory cytokines and their receptors, T/B cells co-stimulation, intracellular signaling, and other targets [16]. Additionally, regenerative medicine, such as stem cell therapy, is being explored as a potential option to repair or replace the damaged salivary and lacrimal glands, which could provide a more definitive solution to the SS.

This review summarizes new treatment strategies for SS, including various clinical drugs, preclinical drugs, and cutting-edge treatment technologies. These advancements can propel progress in new targeted therapies and stimulate new ideas for future SS treatment plans.

## 2 Targeted therapy for SS

**Fig. 1:**
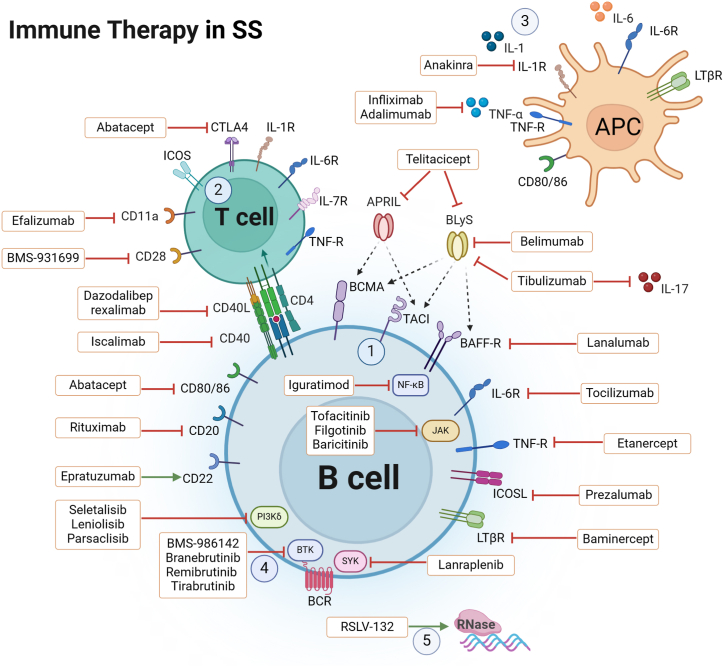
The targets of Sjögren's syndrome treatments ①Targeting B cells; ②Targeting T cells or B/T cells co-stimulation; ③Targeting cytokines and chemokines (or their receptors); ④Small molecules targeting intracellular signaling pathways; ⑤ Other mechanisms of action.

The drugs being evaluated in clinical trials and various preclinical medicines used for SS treatment are highlighted in Table 1. The targets of SS drug therapy are shown in Fig. 1.

**Table 1:** The main potential clinical trial drugs and Preclinical drugs for SS treatment.

Types	Name	Targets/mechanisms	Indications	Status	References
B-cell targeted therapy	Rituximab	Chimeric anti-CD20 monoclonal antibody	Relieving symptoms such as inflammatory arthritis, kidney involvement, vasculitis, severe parotid swelling, pulmonary disease, and mononeuritis multiplex. However, the data available from clinical trials with rituximab are often controversial	Clinical trial	[23], [24], [25], [26], [27], [28], [29], [30]
	Obinutuzumab	Anti-CD20 antibody	Good treatment response to the rituximab resistant SS patients without any symptomatic cross-immunity	Clinical trial	32
	Belimumab	Anti-BAFF monoclonal antibody	Reduced average dryness, ESSDAI, and ESSPRI, restored B cell subsets and their expression of B cell activating factor receptor	Clinical trial	36, 37
	Ianalumab	A novel, defucosylated, human IgG1 anti-BAFF-receptor mAb that targets BAFF receptors	The primary endpoint of ESSDAI was reduced, and improvements were seen across the clinical secondary outcome measures	Clinical trial	[40], [41], [42], [43]
	Telitacicept	A fusion protein comprising a recombinant transmembrane activator, calcium modulator, and cyclophilin ligand interactor receptor fused to the fragment crystallizable (Fc) domain of human IgG	Reducing the ESSDAI score, improving the fatigue status and salivary and lacrimal gland functions	Clinical trial	44
	Epratuzumab	CD22-targeted humanized monoclonal IgG antibody	Improving the fatigue, patient and physician global assessment	Clinical trial	[45], [46], [47]
Small molecule inhibitors	Remibrutinib	Bruton's tyrosine kinase inhibitor	Improving ESSDAI score and unstimulated salivary flow	Clinical trial	49
	Tirabrutinib	Bruton's tyrosine kinase inhibitor	Neither the primary nor secondary endpoints were met in patients with active SS	Clinical trial	51
	Lanraplenib	Spleen tyrosine kinase inhibitor	neither the primary nor secondary endpoints were met in patients with active SS	Clinical trial	51
	Tofacitinib	JAK inhibitor	Improving both symptoms and signs of dry eye syndrome	Clinical trial	[52], [53], [54]
	Baricitinib	Selective JAK1 and JAK2 inhibitor	Improvement in the ESSDAI score and managing different symptoms of SS	Clinical trial	56, 57
Biologics targeting co-signalling molecules	Abatacept	Soluble fusion protein composed of the extracellular domain of CTLA-4 and a fragment of the Fc portion of human IgG1	Improving dry eye, disease activity, rheumatoid factor and IgG levels, fatigue, and health-related quality of life. However, the data available from clinical trials with abatacept are often controversial	Clinical trial	[59], [60], [61], [62]
	Baminercep	lymphotoxin β receptor fusion protein	Baminercept failed to control germinal centers and germinal centers-like structure formation in patients with SS	Clinical trial	65
	Iscalimab	A fully human, pathway-blocking anti-CD40 monoclonal antibody that has been modified with a N297A mutation to render it unable to mediate Fcγ-dependent effector functions	A greater reduction in ESSDAI score	Clinical trial	68
	Prezalumab	A human IgG2 monoclonal antibody against ICOSL and BAFF	The primary endpoint was not achieved	Clinical trial	70
Cytokine targeted therapy	Tocilizumab	Monoclonal antibody against the soluble and membrane-bound IL-6 receptor	There was no improvement observed in the systemic involvement and symptoms	Clinical trial	76
	Adalimumab	TNF-α antibody	Treatment of SS associated with Crohn's disease	Clinical trial	77
	Infliximab	Anti-TNF agent	Not improve dryness symptoms and salivary flow	Clinical trial	78
	Etanercept	Anti-TNF agent	Not improve dryness symptoms and salivary flow	Clinical trial	79
	Anakinra	IL-1 receptor antagonist	The primary endpoint did not show a significant decrease in fatigue among individuals with pSS	Clinical trial	80
	Recombinant human IL-2	IL-2 receptor	Enhance the equilibrium of the immune system by elevating the count of Treg cells and B cells	Clinical trial	81, 82
Other biologics	RSLV-132	A novel, fully human biologic Fc fusion protein that is comprised of human ribonuclease (RNase) fused to the Fc domain of human IgG1	Improvement severe fatigue	Clinical trial	[83], [84], [85]
	RO5459072	A covalent, reversible and selective inhibitor of cathepsin S	No clinically relevant improvement in ESSDAI score (primary endpoint), and no apparent benefit in any of the secondary clinical endpoints	Clinical trial	[86], [87], [88]
Preclinical drugs	Seletalisib	PI3Kδ inhibitor	Improving saliva flow, and reduced autoantibody production	Preclinical trial	89
	Parsaclisib	PI3Kδ inhibitor	Reducing autoreactive B-cells and autoantibody levels, and improving B-cell-mediated antibody-driven salivary gland inflammation	Preclinical trial	90
	AG490/ruxolitinib	JAK inhibitor	Reversing the expression of DNA dioxygenase TET3 in salivary gland epithelial cells	Preclinical tria	91
	Filgotinib	JAK inhibitor	Increasing salivary flow rates and marked reductions in lymphocytic infiltration of salivary glands	Preclinical tria	8
	Cepharanthine	A biscolaurine alkaloid isolated from the plant Stephania cephalantha Hayata	Suppressing interferon-γ-induced CXCL10 expression	Preclinical trial	92
	Fangchinoline	A bisbenzylisoquinoline alkaloid extracted from Stephania tetrandra S	Inhibiting proliferation of neoplastic B cells and alleviating SS-like responses via the Akt/mTOR pathway	Preclinical trial	93
	Iguratimod	Inhibiting the production of inflammatory cytokines	Suppressing plasma cell differentiation and ameliorates experimental Sjögren's syndrome in mice	Preclinical trial	94

ESSDAI EULAR Sjögren's syndrome (SS) disease activity index (ESSDAI), ESSPRI EULAR SS patient reported index, BAFF B-cell Activating factor of the tumor necrosis factor family, JAK Janus kinase, PI3Kδ Phosphoinositide 3-kinase δ, ICOSL Inducible co-stimulator ligand, ten-eleven translocation 3 (TET3), CXCL10 C-X-C motif chemokine 10, protein kinase B (AKT)/mammalian target of rapamycin (mTOR).

### 2.1 Drugs in clinical studies

#### 2.1.1 B cell targeted therapy

According to the EULAR recommendation for SS patients, regimens with monoclonal antibodies targeting B cells may be initiated in severe, refractory systemic disease [17]. The treatment approaches to target the B cell compartment can be summarized as direct depletion, typically with monoclonal antibodies (e.g., rituximab, RTX), indirect depletion via survival cytokine blockade (e.g., belimumab), small molecular inhibitors (e.g., bruton's tyrosine kinase, BTK) [18,19], co-stimulatory blockade agents (e.g. therapeutics targeting CD40L, or inducible co-stimulator) [20], and plasma cell targeting [21]. RTX, a chimeric anti-CD20 monoclonal antibody, is the biological agent of choice in severe extra-glandular manifestations of SS [22]. It can relieve symptoms such as inflammatory arthritis, kidney involvement, vasculitis, severe parotid swelling, pulmonary disease, and mononeuritis multiplex [[23], [24], [25]]. RTX prevented the worsening of salivary gland inflammation by inhibiting the accumulation of class-switched memory B cells [26]. Furthermore, RTX significantly downregulated genes involved in immune-cell recruitment, lymphoid organization alongside antigen presentation, and T-cell co-stimulatory pathways. In the peripheral compartment, RTX downregulated immunoglobulins, autoantibodies, pro-inflammatory cytokines, and chemokines. However, the data available from clinical trials with RTX are often controversial [17]. While some studies have shown positive results, others have found that RTX did not alleviate symptoms or disease activity in patients with SS [[27], [28], [29]]. A single treatment course of RTX led to only modest clinical benefits [30]. Obinutuzumab is another anti-CD20 antibody that is used in the treatment of lymphoproliferative malignancies [31]. Recent studies have investigated the potential of obinutuzumab for patients with SS who are resistant to RTX and have found that around half of the patients responded well to the treatment without any symptomatic cross-immunity [32]. However, further research is needed to identify the specific patient groups that would benefit the most from this treatment.

Belimumab is an anti-BAFF monoclonal antibody that has shown potential as a biotherapeutic drug for autoimmune disorders [33,34]. In SS, belimumab can effectively inhibit rheumatoid factor-positive B cell proliferation [35]. Studies have reported that SS patients treated with belimumab showed a decrease in mean dryness, ESSDAI, and ESSPRI [36]. Belimumab can restore B cell subsets and their expression of BAFF receptors in SS patients [37]. Natural killer cells in the blood and saliva were found to be correlated with a response to belimumab [38]. Combining belimumab with RTX can induce enhanced salivary gland B cell depletion relative to the monotherapies [39]. Ianalumab (VAY736) is a novel, defucosylated, human IgG1 anti-BAFF-receptor mAb that targets BAFF receptors and has shown potential as a therapeutic option for SS patients [40]. It is well tolerated and safe, with no infection increase [41]. The rapid depletion of circulating B cells was observed in ianalumab-treated SS patients [42,43]. Telitacicept is a fusion protein comprising a recombinant transmembrane activator, calcium modulator, and cyclophilin ligand interactor receptor fused to the fragment crystallizable (Fc) domain of human IgG. Telitacicept can inhibit autoreactive B cell maturation and plasma cell differentiation and reduce autoantibody secretion. It can significantly improve the fatigue status of patients and has a specific improvement trend in the salivary and lacrimal gland functions [44]. It showed good safety and tolerability in SS patients, with no severe adverse reactions.

Epratuzumab is a CD22-targeted humanized monoclonal IgG antibody that can down-regulate B cell-receptor signaling. CD22 ligand-binding and signaling domains reciprocally regulate B-cell Ca^2+^ signaling, which is relevant for controlling autoimmunity [45]. Epratuzumab may have clinical benefits in systemic lupus erythematosus patients with associated SS [46]. An open-label phase I/II study of epratuzumab in 16 patients with SS was completed in 2006. The results showed significant improvements in fatigue patient and physician global assessment. It was observed that SS patients have a CD22 over-expression in their peripheral B cells, which was downregulated by epratuzumab [47].

#### 2.1.2 Small molecule inhibitors targeting signaling pathways

BTK is a key signaling node in B cell and Fc receptor signaling. Remibrutinib (LOU064) is a potent, highly selective covalent BTK inhibitor with a promising clinical profile for autoimmune indications [48]. In a phase 2 trial for SS, remibrutinib has demonstrated promising efficacy and safety, significantly improving ESSDAI score and unstimulated salivary flow in SS patients [49]. Tirabrutinib, a small molecule BTK inhibitor, has been approved for treating B-cell malignancies [50]. Although Tirabrutinib was well-tolerated in patients with active SS, neither the primary nor secondary endpoints were met [51]. Lanraplenib is a potent and selective inhibitor of spleen tyrosine kinase, which is a critical regulator of signaling in various immune cell types such as B-cells, monocytes, and macrophages. The use of lanraplenib was found to be well-tolerated in patients with active SS. However, neither the primary nor the secondary endpoints were met [51]. Tofacitinib is a novel oral JAK inhibitor that can counteract IL-6 overexpression induced by deficient autophagy [52]. Studies have confirmed that tofacitinib improves symptoms and signs of dry eye syndrome [53,54]. Autophagy-deficient three-dimensional-acini reproduced the findings observed in the labial salivary gland from SS patients, showing increased expression of pro-inflammatory markers such as IL-6, which was reversed by tofacitinib [52]. Tofacitinib exhibited a reasonable safety profile and was well tolerated. Baricitinib is an oral selective inhibitor of JAK1 and JAK2, which has demonstrated significant efficacy in rheumatoid arthritis (RA) patients [55]. Baricitinib has shown significant improvement in the ESSDAI score and could potentially aid in managing different symptoms of SS, including arthritis, interstitial lung disease, constitutional symptoms, hematological involvement, and skin rash [56]. Currently, a multi-center, open-label, randomized study is being conducted to explore the efficiency and safety of baricitinib in treating active SS patients [57].

#### 2.1.3 Biologics targeting Co-signaling molecules

Abatacept is a soluble fusion protein composed of the extracellular domain of CTLA-4 and a fragment of the Fc portion of human IgG1. Abatacept binds more strongly to CD80/CD86 than CD28, preventing T-cell activation [58]. Studies have confirmed that abatacept is effective, safe, and well-tolerated for treating SS, resulting in improved disease activity, rheumatoid factor, IgG levels, fatigue, and health-related quality of life in SS [[59], [60], [61]]. However, recent research has found that the abatacept treatment did not have significant clinical efficacy in patients with moderate-to-severe SS [62]. Baminercept is a lymphotoxin β receptor fusion protein, which can block the lymphotoxin-αβ/LIGHT axis, alter lymphocyte trafficking, and inhibit the whole blood IFN signature [63]. Lymphotoxin-beta receptor (LTβR) blockade may have therapeutic potential for treating SS [64]. In a phase II study, treatment with baminercept failed to significantly improve glandular and extra-glandular disease in SS patients, despite evidence from mechanistic studies showing that it blocks LTβR signaling [65]. Iscalimab, a fully human, pathway-blocking anti-CD40 monoclonal antibody that has been modified with a N297A mutation to render it unable to mediate Fcγ-dependent effector functions, can blockade the CD40^−^CD40L pathway, inhibiting SS-related pathology [66,67]. Compared to the placebo, iscalimab treatment by intravenous resulted in a more significant reduction in ESSDAI score [68]. The prezalumab is a human IgG2 monoclonal antibody against ICOSL and BAFF and has been shown to benefit lupus patients with arthritis [69]. A phase II RCT of prezalumab did not meet the primary endpoint for SS (ACR abstract NCT02334306) [70]. Acazicolcept is an Fc fusion protein of the human ICOSL and a variant immunoglobulin domain (vIgD™) designed to inhibit the CD28 and ICOS T cell costimulatory pathways. Azicolcept may be an effective therapeutic agent for RA and psoriatic arthritis by co-inhibiting inducible co-stimulators and CD28 signaling [71]. The role of acazicolcept in SS needs further research and validation.

#### 2.1.4 Cytokine targeted therapy

Tocilizumab, a monoclonal antibody against the soluble and membrane-bound IL-6 receptor, has demonstrated efficacy and safety in RA, systemic sclerosis, and systemic lupus erythematosus [72,73]. Some case studies have confirmed the potential of tocilizumab for treating SS and its complications [74,75]. However, a study has demonstrated that tocilizumab did not improve systemic involvement and symptoms in SS patients [76]. Adalimumab is a human monoclonal TNF-α antibody that works by blocking the effects of TNF-α. It has been reported that adalimumab has successfully treated SS associated with Crohn's disease [77]. TNF-α blockers (infliximab, etanercept) did not improve dryness symptoms and salivary flow in SS patients [78,79]. A study was conducted to assess the effectiveness of anakinra, an IL-1 receptor antagonist, in SS patients based on the hypothesis that IL-1 may be involved in fatigue. The primary endpoint of the two fatigue scores was not met [80]. Low-dose IL-2 was effective and well-tolerated for SS patients, which can restore immune balance by increasing the number of Treg cells and CD24^high^CD27^+^ B cells [81,82]. Herein, the biologics aimed at pro-inflammatory cytokines have demonstrated limited efficacy in treating SS. Clinical trials assessing the effectiveness of cytokine-related inhibitors in SS are still pending.

#### 2.1.5 Other biologics under Evaluation for SS treatment

RSLV-132 is a novel, fully human biologic Fc fusion protein that is comprised of human ribonuclease (RNase) fused to the Fc domain of human IgG1. RSLV-132 targets immune complexes and renders them dysfunctional through the breakdown of RNA [83]. Research has confirmed that RNase can enhance Fcγ receptor activation by promoting the formation of immune complexes containing Ro/SSA or La/SSB [84]. In a randomized clinical trial, the administration of RSLV-132 has been found to improve severe fatigue in patients with SS [85]. RO5459072 is a covalent, reversible, and selective inhibitor of cathepsin S developed for the treatment of SS [86]. Inhibition of cathepsin S by RO5459072 could diminish T cell and associated monokine responses towards relevant autoantigens in SS patients [87]. However, there was no clinically relevant improvement in ESSDAI score (primary endpoint) or apparent benefit in favor of cathepsin S inhibitor RO5459072 in any of the secondary clinical endpoints [88].

### 2.2 Drugs in preclinical studies

The latest research findings in drugs in preclinical studies for SS have shown promising developments. The phosphatidylinositol 3-kinase delta (PI3Kδ) pathway is a novel therapeutic target for SS. Seletalisib, a selective inhibitor of PI3Kδ, has shown promise in a murine model of focal sialoadenitis, a condition characterized by the inflammatory destruction of salivary glands. Seletalisib effectively reduced the accumulation of lymphocytes and plasma cells within the glands. Furthermore, treatment with seletalisib significantly affected the production of lymphoid chemokines and cytokines, improved saliva flow, and reduced autoantibody production [89]. Parsaclisib, a potent and selective PI3Kδ inhibitor, has been found to reduce autoreactive B-cells and autoantibody levels and significantly improve B-cell-mediated antibody-driven salivary gland inflammation in SS [90]. JAK/STAT signaling potentially affects DNA methylation/hydroxymethylation alterations in SS. JAK inhibitors (AG490 and ruxolitinib) have been shown to reverse the expression of DNA dioxygenase ten-eleven translocation 3 (TET3) in salivary gland epithelial cells [91]. JAK inhibitor (filgotinib) treated mice exhibited increased salivary flow rates and marked reductions in lymphocytic infiltration of salivary glands [8]. Cepharanthine, a biscolaurine alkaloid isolated from the plant Stephania cephalantha Hayata, is effective in suppressing IFN-γ-induced CXCL10 expression by inhibiting JAK-STAT signaling in human salivary gland ductal cells [92]. Fangchinoline, a bisbenzylisoquinoline alkaloid extracted from Stephania tetrandra S, can inhibit the proliferation of neoplastic B cells and alleviate SS-like responses via the Akt/mTOR pathway [93]. The latest research confirms that iguratimod bound to the regulatory factor for B cells (TEC kinase) and promoted its degradation through the autophagy-lysosome pathway in BAFF-activated B cells, directly inhibiting TEC-regulated B cells function [94]. It is worth noting that preclinical drugs are in the early stages of development. Before conducting clinical trials involving human participants, preclinical studies are crucial for determining the safety and efficacy of drugs.

### 2.3 Cutting-edge technology therapy

Cutting-edge technologies are being developed for the treatment of SS, including gene therapy, tissue engineering, and cell-based therapy [95]. Gene therapy involves using viral or non-viral vectors to transfer genes into residual cells of atrophied glands to promote saliva secretion [96,97]. Genetically engineered drugs, such as adenoviral vectors expressing the IL17R: Fc fusion protein and AQP1-encoding adenoviral vector, are being developed to help avoid systemic immune suppression and severe side effects and have shown great potential for further development as a gene therapy for SS. In addition, modulating the alternative splicing of exons in FoxP3 mRNA by gene editing via CRISPR/CAS9 may become a promising strategy for SS therapy [98]. Vaccines such as the CD40 DNA and anti-IFN-α vaccination strategies have also shown efficacy in treating SS [99,100]. For note, mesenchymal stem cells (MSCs) transfusion can differentiate into salivary epithelial cells, restore salivary gland secretory function, and suppress autoimmunity in SS by inducing Tregs suppressing Th1, Th17, and T follicular helper cell responses [101]. MSC injections can lead to a decrease in inflammatory cytokines and an increase in anti-inflammatory cytokines [102]. Delivery of MSCs can improve tear production in a mouse model of SS [103]. MSCs can ameliorate SS by the multifaceted regulation of immune responses and regenerative functions [104,105]. Human umbilical cord MSCs have been found to be capable of inducing CD4^+^Foxp3^+^T cells and effectively interfering with the autoimmune attack during SS [106]. Injection of adipose-derived MSCs into the lacrimal gland can improve subjective dry eye symptoms, especially tear film stability in patients with aqueous dry eye disease due to SS [107]. In recent years, tissue engineering-based organ regeneration has emerged as a potential alternative lacrimal or salivary gland failure treatment. Several strategies have been considered to regenerate, repair, or replace salivary glands [108,109]. Bioengineered salivary gland organoids may be the solution for severely damaged salivary glands unsuitable for cell therapy. However, successful engraftment of these organoids requires a favorable environment characterized by sufficient blood vessel supply and suppression of excessive immune reactions. Cell-based therapy is a promising candidate approach to establishing such an environment. While cell-based therapies are still in the developmental stage and face challenges such as complex manufacturing processes, high labor costs, and high cost of clinical application, they hold great potential for treating salivary gland dysfunction with further research.

## 3 Concluding remarks and future perspectives

Previous studies on the treatment of SS mainly focused on symptomatic treatment to prevent gland injury and inhibit the activity of systemic diseases. Immunosuppressive agents, glucocorticoids, and biological agents are primarily used to control disease development and reduce disease activity. Currently, the exact cause of SS has yet to be discovered, making it challenging to create a unified treatment plan. There is a need for further in-depth discussion on how to optimize clinical treatment. This study describes novel agents for specific molecular pathways involved in SS, such as target B cell hyperactivity, targeting B cell and T cell co-stimulation pathways, and targeting pro-inflammatory cytokines. The novel therapeutic strategies for SS, including gene therapy, tissue engineering, and cell-based therapy, were also explored. Such advancements promise to propel the development of targeted therapies and spark innovative ideas for treatment paradigms in SS.

With ongoing research on the pathogenesis of SS, the potential therapeutic targets have been identified, and targeted drugs have shown promising therapeutic effects. Improving the staging process for identifying early disease and discovering novel biomarkers and genetic profiles in SS patients is crucial to addressing unmet clinical needs. There is a rapid emergence of innovative technologies that have the potential to cause significant changes. Immune cells are being engineered to recognize and respond to disease states, acting as a "living drug” when transferred into SS patients. Immunoengineering could aid in diagnosing SS, immunophenotyping for patient-stratification purposes, and developing in vitro models to study autoimmunity. Advanced technologies offer promising avenues for treating SS, and ongoing research in the above field is expected to lead to further breakthroughs.

Although there has been progress, there is still a lack of specific drugs for treating SS, and existing drugs still require systematic clinical research. Several key issues need to be addressed to enhance SS treatment prospects further. (1) Predictors of response to different treatments. (2) Biomarkers of response to different biological agents. (3) Identify predictors of SS patients with different systemic complications. (4) Evaluation and standardization of disease activity/outcomes reported by SS patients. (5) Adoption of better and more sensitive disease activity assessment tools. (6) Control background medication to avoid diluting the efficacy of experimental drugs due to multiple drugs. (7) Optimize clinical study design and endpoints and maximize the likelihood of new drug approval. Some patients with advanced diseases may have irreversible glandular function when enrolled, which may not better reflect the efficacy of targeted drugs.

Focusing on specific immune cells that play a role in disease progression, such as disease-specific T cells and B cells, could be a practical treatment approach for SS patients. While some targeted drugs have shown promise in experiments, only a few have been used in clinical settings, and the process is usually lengthy. It is essential to develop a safe and effective treatment plan with fewer side effects by combining existing drugs based on a comprehensive assessment of patients' conditions and with the help of a multidisciplinary approach. The current treatment plan has limited evidence of involvement in SS and its systemic complications. However, by exploring the etiology and pathogenesis of SS, molecular theoretical support has been provided for the precise treatment of SS. Hopefully, with the promotion of targeted therapeutic drugs in clinical practice, disease progression can be effectively controlled in the early stage, and the long-term prognosis and quality of life of SS patients can be improved.

## Funding

This research was supported by 10.13039/501100001809National Natural Science Foundation of China (82074341), Scientific Research Projects for High-level Talents of Yunnan University of Chinese Medicine (2019YZG01), Young Top-Notch Talent in 10,000 Talent Program of Yunnan Province (YNWR-QNBJ-2019-235), Postdoctoral Fellowship Program of CPSF (GZB20230665, GZC20232376), the China Postdoctoral Science Foundation (2024M752911, 2024M752910), the Research Project of Zhejiang Chinese Medical University (2023RCZXZK51, 2023RCZXZK50).

## Ethics declaration

Review and/or approval by an ethics committee as well as informed consent was not required for this study because this literature review only used existing data from published studies and did not involve any direct experimentation/studies on living beings.

## Data availability statement

No data was used for the research described in the article.

## CRediT authorship contribution statement

**Ting Zhao:** Writing – review & editing, Writing – original draft, Supervision, Conceptualization. **Runrun Zhang:** Validation, Methodology, Formal analysis. **Zhaofu Li:** Visualization, Supervision, Resources, Investigation, Conceptualization. **Dongdong Qin:** Validation, Resources, Funding acquisition, Formal analysis. **Xinchang Wang:** Supervision, Resources, Methodology, Funding acquisition, Conceptualization.

## Declaration of competing interest

The authors declare that they have no known competing financial interests or personal relationships that could have appeared to influence the work reported in this paper.
